# Supplementary material for: Chromothripsis during telomere crisis is independent of NHEJ, and consistent with a replicative origin
Source: Genome Res. 2019 May;29(5):737–49. doi: 10.1101/gr.240705.118 (PMC6499312; doi:10.1101/gr.240705.118)
Supplement: Supplemental Material [file supp_gr.240705.118_Supplemental_file_1.zip › contigs/annotated_contigs/DB48/contig.2.DB48_length_1014_mean_cov_19.9063116371.docx]

**DB48_length_1014_mean_cov_19.9063116371**

```
TTCTGAATTTAGTGGTAAAAGGGGGTGTGGTTTATGGGTCTTTTTAAGCTTCACCATAAGGGGTTTTAATTTGAAATTGAAAAGGAGAC
 >chr7:51633651-51634184 - E=1e-293 p=8e-03
ATGTCCATGCAGCTAGCGGCAGGCAAGACCAACTTCACACGTGGGAAGTGGAAGGTCTCGGTGGTGGCCAAGGAACGAGAGTGGCTGTG

GGGGCTGGGGACTCTTACTTACCATTTGGGGTCCCAGCAGCCTGTTGTGTCCAGCAGCCATAGGTACCCAGTTGTGCGTTTAATAGGCC

CCTGGCTGTTTTTAAAAGGTACAAACTTTAGTCAGCTCTGGACAAGCAATGCCAACCGCTGGCTTTGACCTTAGCCTGTGAGGTGAGAC

TGAAATGAGATGAAAGGCTTGTCTCAAAACAGACCACAGGCTTTTGAGCCAATGCCATGGGAACTATTTTTGCTCCGTTTAAAATGTGT

TTTCAGCCAGGACTGCAGGCAGGGGCCTTAAACAAGCACGGTGGTTCCTTTACAGGAGGGCTGCGGAGACCCTGCCACTGAAGTT|CCT
                                                                                      >c
|CCAGTCAGTGGGGGAAGGGTAGATGTTGGATGCCACCCCCATAGGGCAGCTCATTCTCCCCTCTTTGGGAGGGTGAGTGGGTAGCTAG
hr7:51634438-51634922 + E=4e-279
AGAAAGGTGGAGATGTGATCCTCCTAGGCTCTTCCTAACTCTGGAAGTGTGAGGTCACTGGGAAGCTTGTTGGAAATGCAGGTTGTCTG

GTCCCACCCAGATGAATGGAATGAGAAATTCAGAGAGTGGGCTTGGTAACCCATGCTTGAACAAGCCTGCAAGATGATGCAGGTGCAGA

GCAGAACTTCCCAACCTCGGTACAGTTGACATTTTGTGGACATAATTCTTTACTCAGTGTGTGTGTGTGTGTGTGTGTGTGTGTGTCCT

GGATATTGTAGAATGTTTAGCAGCATCTCTGGTCCCTACCCACTAGATGCCAGTAGTACCCTTGACAACCAAAAGTGTTTCCAGCCATT

GCTAAGTATCCCCAAGGGGGAAAAGTCACCTACAGAA
```
